# Preparation and characterization of *Allium cepa* extract coated biochar and adsorption performance for hexavalent chromium

**DOI:** 10.1038/s41598-023-48299-8

**Published:** 2023-11-27

**Authors:** James Friday Amaku, Raymond Taziwa

**Affiliations:** Department of Applied Science, Faculty of Science Engineering and Technology, Walter Sisulu University, Old King William Town Road, Potsdam Site, East London, 5200 South Africa

**Keywords:** Environmental sciences, Chemistry, Materials science

## Abstract

The elimination of hazardous metal ions from contaminated water has been an important procedure to improve the quality of the water source. Hence, this study presents the fabrication of *Allium cepa* extract-coated biochar for the elimination of Cr (VI) from wastewater. The synthesized biochar (SBCH) and modified biochar (BMOJ) were characterized by making use of FTIR, BET, XRD, TGA and SEM. Optimum Cr (VI) removal was achieved at solution pH 2, 0.05 g adsorbent dosage and 180 min agitation period. The adsorptive removal of Cr (VI) onto SBCH and BMOJ followed the pseudo-second-order kinetic model with a satisfactory sum of square residuals (SSR) of 3.874 and 5.245 for SBCH and BMOJ, respectively. Meanwhile, Freundlich isotherm was found to best describe the uptake of Cr (VI) SBCH and BMOJ. Experimental data showed an adsorption capacity of 37.38 and 25.77 mg g^−1^ and a maximum efficiency of 85.42% and 51.63% for BMOJ and SBCH, respectively. BMOJ also showed good antioxidant characteristics. Thermodynamic data revealed that the uptake of Cr (VI) onto the SBCH and BMOJ was an exothermic and endothermic (ΔH: SBCH =  − 16.22 kJ mol^−1^ and BMOJ = 13.74 kJ mol^−1^), entropy-driven (ΔS: SBCH = 40.96 J K^−1^ mol^−1^ and BMOJ = 93.26 J K^−1^ mol^−1^) and spontaneous process. Furthermore, BMOJ demonstrated excellent reusability and promising characteristics for industrial applications.

## Introduction

In accordance with the findings, one of the most important issues confronting humanity is the ongoing contamination of the environment, which has harmed the aquatic ecosystem^1^. The continuous contamination of the waterbodies may be attributed to natural disasters, anthropogenic activities, rapid economic and social growth, population explosion and technological advances^2–5^. Among these water contaminants, Chromium (Cr) has been observed to pose a severe challenge to man and his environment. Electroplating, tannery, steel, dye, cement, metallurgy, metal plating, and textile industries are known to discharge large amounts of Chromium ions into bodies of water^2^. In the aqueous phase, Cr exists in various oxidation states, however, the hexavalent and trivalent forms of Cr are the most dominant^6^. The trivalent form of Cr is more stable, less hazardous, and plays a vital role in the regulation of glucose metabolism by insulin^7^. On the other hand, hazardous Cr (VI) exist as oxyanions and their species (HCrO_4_^−^ Cr_2_O_7_^2−^, H_2_Cr_2_O_7_, and CrO_4_^2−^) are pH dependents^8^. Hexavalent chromium has been linked to organ damage, cancer, and mutation in those who have been exposed to high levels of this dangerous heavy metal ion^9^. The biochemical (interaction with DNA molecules) and physicochemical characteristics (oxidative potential, ionic size, and high solubility) of Cr (VI) make this water contaminant very harmful and difficult to remove from the water bodies. Stakeholders such United States Environmental Protection Agency (USEPA) and the World Health Organization (WHO) recommended 0.05 mg dm^−3^ and 0.1 mg dm^−3^ as the threshold concentration for Cr in portable and surface water^10–12^.

To maintain a clean, healthier, and less harmful aquatic ecosystem, contaminants such as Cr (VI) must be removed from the water bodies. To achieve this, different techniques such as adsorption^13–17^, biodegradation^18^, osmosis^19^, ion exchange^20^, electrocoagulation^21^, chemical precipitation^22^ photocatalysis^23^ and membrane filtration^24^ and have been used for the treatment Cr (VI) contaminated wastewater. With the exception of the adsorption technique, the previously mentioned water treatment techniques are known to have drawbacks such as poor efficiency at low analyte concentration, generation of secondary water contaminants and high-cost operation. Comparatively, adsorption has demonstrated exceptional capacity for the remediation of Cr (VI) by making use of adsorbents such as layered double hydroxides^25^, silica^26^, Graphene oxide^27–29^, tea waste^30^, manganese oxides^31^, activated carbon^32–34^, chitosan^35^, biochar^36,37^, clay^38^, MgO^39^, microplastics^40^, nanocomposite^41^, groundnut shell^42^, hydrogel^43^, water hyacinth roots^44^, pomelo fruit peel^45^ and Zr-MOF^46^. However, the value of an adsorbent is a function of its removal potential, reusability tendency, efficacy at low analyte concentration and techno-economic implications^47,48^. Most of the adsorbents that have been employed for the removal of Cr (VI) have some degree of limitations^49^. This shows the need to design much more efficient adsorbents with exceptional potential for Cr (VI) sequestration.

Biochar is a carbon-rich material produced using the pyrolytic process^50,51^. This process is thought to entail a thermochemical breakdown of biomass at relatively high temperatures in an oxygen-limited environment^52^. They are prepared from plant materials or animal waste, they are porous and have been reported to have properties that support wastewater treatment^50,53^. However, there are limitations to the application of biochar in water treatment and this can be harnessed by modifying the surface with the right material and method^54^. To design a biochar that is fit for environmental remediation practices, it is important to employ modifiers that are cheap, easy to source and environmentally friendly^55^. Modifiers of organic origin are often sourced from plants. Due to the lack of processing and storage equipment in Africa, perishable vegetables have constituted a major source of waste^56^. Hence, the conversion of agricultural waste to value-added products for environmental remediation is of great benefit^57^. Plant extracts constitute phytochemicals that are good reductants. Speaking of perishable vegetables, *Allium cepa* extract contains a reasonable amount of phytochemicals with good anti-oxidative characteristics^58–60^. It has been reported that *Allium cepa* extract contains phytochemicals such as allicin, fisetin, quercetin, anthocyanins, and kaempferol amongst others^61–66^. Hence, the application of *Allium cepa* extract as biochar-modifier, may enhance the capacity biochar modified adsorbent for Cr (VI) sequestration from aqueous solution.

In this report, a novel adsorbent was fabricated from biochar sourced from *Annona muricata* petal and *Allium cepa* extract. The effectiveness of the adsorbents in eliminating Cr (VI) from aqueous solution was investigated. This was accomplished by examining the influence of initial PH, agitation period, adsorbent dose, and initial sorbate concentration on the adsorptive removal of Cr (VI). Thermodynamics, kinetic and isotherm of adsorbate uptake by the prepared adsorbent were depicted to provide detailed insight into the process of adsorbate removal. The possible reuse of the adsorbent for Cr (VI) was also looked into.

## Experimental

### Material and chemicals

The *Allium cepa* L were collected from the dry garbage container at the Ahia Eke community market, Umuahia, Abia State, Nigeria (5° 30′ 43.0" N and 7° 31′ 48.0" E). *Annona muricata* petals were collected at the National Root Crops Research Institute (NRCRI) in Umudike, Abia State, Nigeria. The adsorption experiments employed high-purity analytical grade chemical reagents such nitric acid (HNO_3_, 98%), sulphuric acid (H_2_SO_4_, 98%), potassium dichromate (K_2_Cr_2_O_7_, 99.0%), sodium hydroxide (NaOH, 99.99%), sodium chloride (NaCl, > 95%) and hydrochloric acid (HCl, 99.9%) were obtained from Sigma–Aldrich and used without supplementary purification.

### Extraction of modifier

About 500 g of *Allium cepa* was washed and pulverized using an electric blender. The mixture was filtered to remove the pulp, thereafter, the juice was vacuum oven-dried at 50 °C. The concentrate of the *Allium cepa* was then stored for further use.

### Adsorbent preparation

*Annona muricata* petals collected from NRCRI were washed to remove dirt, air-dried and pulverized to a fine powder using an electric blender. The pulverized petals were then passed through sieves with a mesh size of 2000 μm. The sieved pulverized petals were pyrolyzed at a temperature of 350 °C for 30 min. The combustion procedure was performed in a limited air-supplied environment inside the tubular furnace. Following that, the resulting biochar (SBCH) was pulverized and sieved through a 500 μm mesh sieve. The sifting was done to get uniform biochar for instrument characterization. About 0.025 g of *Allium cepa* concentrate was redissolved in a solution (25 mL) containing 5 g of SBCH. The mixture was thereafter stirred to dryness and the black product obtained was stored for future use.

### Characterization

The implication of system temperature (thermogravimetric analysis/differential thermal analysis (TGA/DTA) on SBCH and BMOJ were investigated using a PerkinElmer simultaneous thermal analyzer STA6000 instrument, USA. Surface morphologies of SBCH and BMOJ were studied by scanning electron microscopy (SEM) using a ZEISS ultra plus, USA unit. Prior to micrograph acquisition, SBCH and BMOJ were sputtered with a thin electric conductive gold coating to avoid charging effect. SBCH and BMOJ were subjected to Fourier transformation infrared spectroscopy (FT-IR) using Nicolet-870 spectrophotometer US. The X-ray powder diffraction (PXRD) patterns of SBCH and BMOJ were acquired using XRD Bruker D8 Advance powder X-ray diffraction equipped with a monochromatic X-ray of Cu K_α1_. Specific surface area, pore diameter and pore volume of degassed (overnight at 105 °C) SBCH and BMOJ were estimated via the Nitrogen (N_2_) physisorption experiments of Brunauer–Emmett–Teller (BET) and Barrett-Joyner-Halenda (BJH) analysis (Micromeritics Instruments Corp., Ltd., USA).

### pH point of zero charge (pH_PZC_).

Using the solid addition method, the effect of pH on the surface charge of SBCH and BMOJ was investigated^67,68^. Briefly, in eleven Erlenmeyer flasks, 0.1 g of SBCH or BMOJ were suspended for 24 h in a pH 2–12 pre-adjusted NaCl (50 mL of 0.1 mol dm^3^) solution. Thereafter, the pH_PZC_ of SBCH and BMOJ was determined by extrapolating the resultant pH versus the inception pH of the mixture^69^.

### Batch adsorption

The adsorption capacity of SBCH and BMOJ was investigated using batch adsorption method with solutions containing varied concentrations of Cr (VI) ions. Prior to the adsorption step, a working solution of Cr (VI) was prepared from the stock solutions (1000 mg dm^−3^, 2.83 g of K_2_Cr_2_O_7_/dm^3^). About 0.05 g of SBCH or BMOJ were correctly weighed into 25 cm^3^ (100 mg dm^−3^) of Cr (VI) solutions adjusted to optimum pH (2.0) and agitated at 120 rpm for 180 min in a thermostatic shaker at room temperature. The influence of initial pH, solution temperature, initial concentration, sorbent dose, and agitation time was assessed. Hexavalent chromium equilibrium concentration was determined using an Ultraviolet–visible spectrophotometer at 540 nm. Finally, the uptake potential and the adsorption efficiency of SBCH and BMOJ were estimated using Eqs. (1) and (2).

1 $${q}_{eq}=\left(\frac{{C}_{i}-{C}_{eq}}{m}\right)V$$

2 $$\% adsorbed=\left(\frac{{C}_{i}-{C}_{eq}}{{C}_{i}}\right)\times 100$$

where C_i_ denotes the initial Cr (VI) concentration (mg dm^−3^), C_eq_ denotes the equilibrium Cr (VI) concentration (mg dm^−3^), m denotes the adsorbent mass (g), and V denotes the volume of adsorbate solution (dm^3^). After loading Cr (VI) onto the SBCH and BMOJ, adsorption–desorption assessments were performed to renew the SBCH and BMOJ for further recycling. Initially, the loaded adsorbents (Cr-SBCH and Cr-BMOJ) were desorbed in 0.5 M NaOH for 12 h at 25 °C with constant shaking. Following that, SBCH or BMOJ were rinsed with a diluted HCl solution to renew the active adsorption sites. To recover the uptake potential of both SBCH and BMOJ in the following adsorption cycle, the acid-rinsed SBCH and BMOJ were then washed with distilled water to neutralize the surface, and the procedure was thereafter repeated four times for SBCH and BMOJ regeneration. Meanwhile, the methods were performed in accordance with the relevant guidelines/regulations/legislation as stipulated.

### Kinetics and isotherm models

Data acquired from the contact time experiment were fixed into Elovich, intraparticle diffusion, pseudo-first order and pseudo-second order kinetics models in the R-statistical interface using their appropriate nonlinear equations, as shown in Table 1. On the other hand, data collected from the effect of concentration experiment was fixed into Freundlich and Langmuir isotherm using their nonlinear equation supplied in Table 2.

**Table 1:** Kinetics models investigated for the adsorption of Cr(VI) onto SBCH and BMOJ.

Kinetic models	Equations	Parameters	References
Pseudo-first order	$\frac{{dq}_{t}}{{d}_{t}}={k}_{1}\left({q}_{e}-{q}_{t}\right)$	${q}_{e}{,k}_{1}$	^70^
Pseudo-second order	$\frac{{dq}_{t}}{{d}_{t}}={k}_{2}{\left({q}_{e}-{q}_{t}\right)}^{2}$	${k}_{2},{q}_{e}$	^71^
Weber–Morris intraparticle diffusion	$\frac{{dq}_{t}}{{d}_{{t}^{-0.5}}}={k}_{id}$	${k}_{id,} l$	^72^
Elovich	$\frac{{dq}_{t}}{{d}_{t}}=\alpha exp\left(-\beta {q}_{t}\right)$	$\alpha , \beta$	^73^

*k*_*1*_ pseudo-first order rate constant (min^−1^), *k*_*2*_ pseudo-second order rate constant (g mg^−1^ min^−1^), *k*_*id*_ intraparticle diffusion rate constant (mg g^−1^ min^0.5^), *l* is a constant related to the boundary layer thickness (mg g^−1^), *q*_*t*_ quantity of adsorbate adsorbed at time t (mg g^−1^), *q*_*e*_ quantity of adsorbate adsorbed at equilibrium (mg g^−1^), *α* adsorption rate constant (mg g^−1^ min^−1^), *β* desorption rate constant (g mg^−1^), *Ceq* the concentration of Cr(VI) in solution at equilibrium (mg dm^−3^).

**Table 2:** Isotherm models used to describe the uptake of Cr(VI) onto SBCH and BMOJ.

Isotherm model	Equation	Parameters	References
Langmuir	${q}_{e}=\frac{{q}_{max}{bC}_{e}}{1+b{C}_{e}}$	${q}_{max},$ b	^74^
Freundlich	${q}_{e}={K}_{F}{C}_{e}^{1/n}$	${k}_{F}, n$	^75^

*q*_*eq*_ adsorption capacity (mg g^−1^) of SBCH or BMOJ, *C*_*eq*_ equilibrium concentration of Cr(VI) in solution (mg dm^−3^), *q*_*max*_ maximum monolayer capacity (mg g^−1^) of SBCH and BMOJ, *b* Langmuir isotherm constant (dm^3^ mg^−1^), *K*_*F*_ Freundlich isotherm constant (mg g^−1^) (dm^−3^ mg^−1^), *n* adsorption intensity.

### Antioxidant assay

The antioxidant activity of SBCH and BMOJ was determined using the DPPH test. A typical procedure involved contacting 0.5 cm^3^ of DPPH (0.3 mM) solution with various doses of SBCH and BMOJ (25, 50, 100, 200, and 400 g cm^−3^). SBCH and BMOJ serve as radical hunters, while DPPH serves as a radical generator. The reaction mixture was then incubated for 30 min in a dark environment at room temperature. The % change in absorption wavelength at 517 nm was utilized to track radical concentration^76,77^. On the other hand, the modified method of Chew and Lim (2018) was used to determine the ferric-reducing antioxidant potential (FRAP) of SBCH and BMOJ^78^. Briefly, about 1 cm^3^ of SBCH or BMOJ (25, 50, 100, 200, and 400 g cm^−3^), 2 cm^3^ of potassium phosphate buffer (0.1 M, pH 6.6), and 1% (m/v) potassium ferricyanide (2.5 cm^3^) were contacted. The mixtures were incubated at 50 °C for 20 min before being treated with 2.5 cm^3^ of 10% (m/v) trichloroacetic acid. To increase colour development, 2.5 cm^3^ of deionized water and 0.5 cm^3^ of 0.1% (m/v) ferric chloride (0.5 cm^3^) were added to 2.5 cm^3^ of the reaction mixtures, and the solutions were incubated at 28 °C for 30 min. The absorbance readings were measured at 700 nm. The percentage inhibition (% I) of the DPPH and the percentage of FRAP in gallic acid equivalents were estimated using Eqs. (3) and (4) respectively.

3 $$I\%=\frac{\left({Absorbance}_{control}-{Absorbance}_{Sample}\right)}{{Absorbance}_{control}}\times 100$$

4 $$\% FRAP=\frac{{Absorbance}_{sample}}{{Absorbance}_{Gallic acid}}\times 100$$

### Data analysis

R statistical computing environment, Microsoft Excel and Origin Pro 8 were employed to process the experimental data acquired in this study^79^.

## Results and discussion

**Figure 1:**
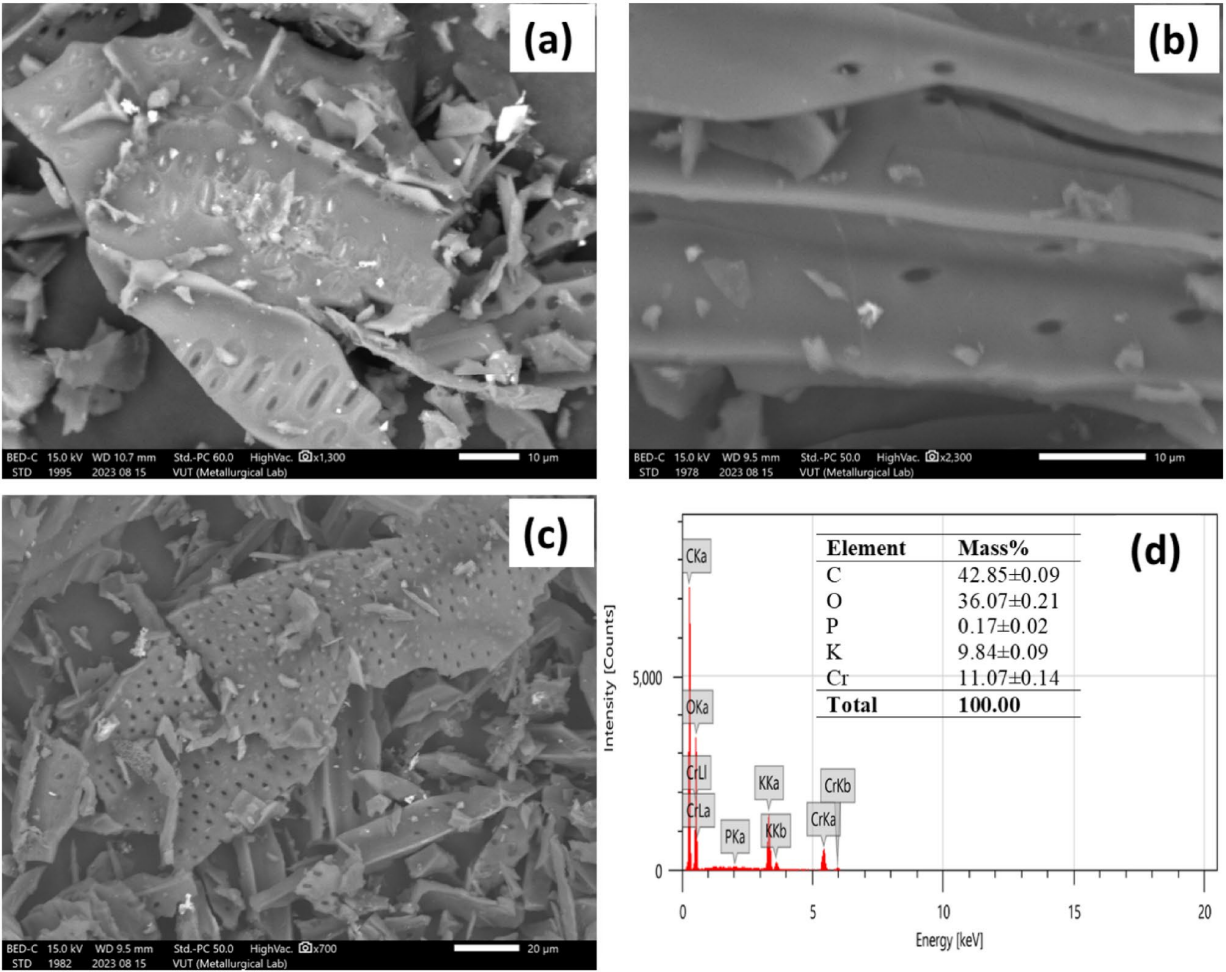
SEM micrographs of (**a**) SBCH, (**b**) BMOJ, (**c**) BMOJ-Cr and (**d**) EDX of BMOJ-Cr.

Figure 1 illustrates the SEM images of SBCH, BMOJ and BMOJ-Cr. The brittle honeycomb skeletal structure of the pristine and modified biochar exhibited irregular layered shapes sustaining some internal pores and cracks. The pyrolytic process that involves the volatilization of organic compounds as a result of the decomposition of more components may be responsible for the formation of deep channels with well-defined clear pores and vesicles. The well outline pores may influence the pore volumes, pore diameter and surface areas of SBCH and BMOJ. Figure 1b, shows a porous smooth surface, this reflects the implication of coating the biochar with *Allium cepa* juice extract. Finally, the EDX spectrum of the spent BMOJ (BMOJ-Cr) clearly showed the incorporation of Cr, alongside other elements, as evidence of Cr (VI) adsorption.

**Figure 2:**
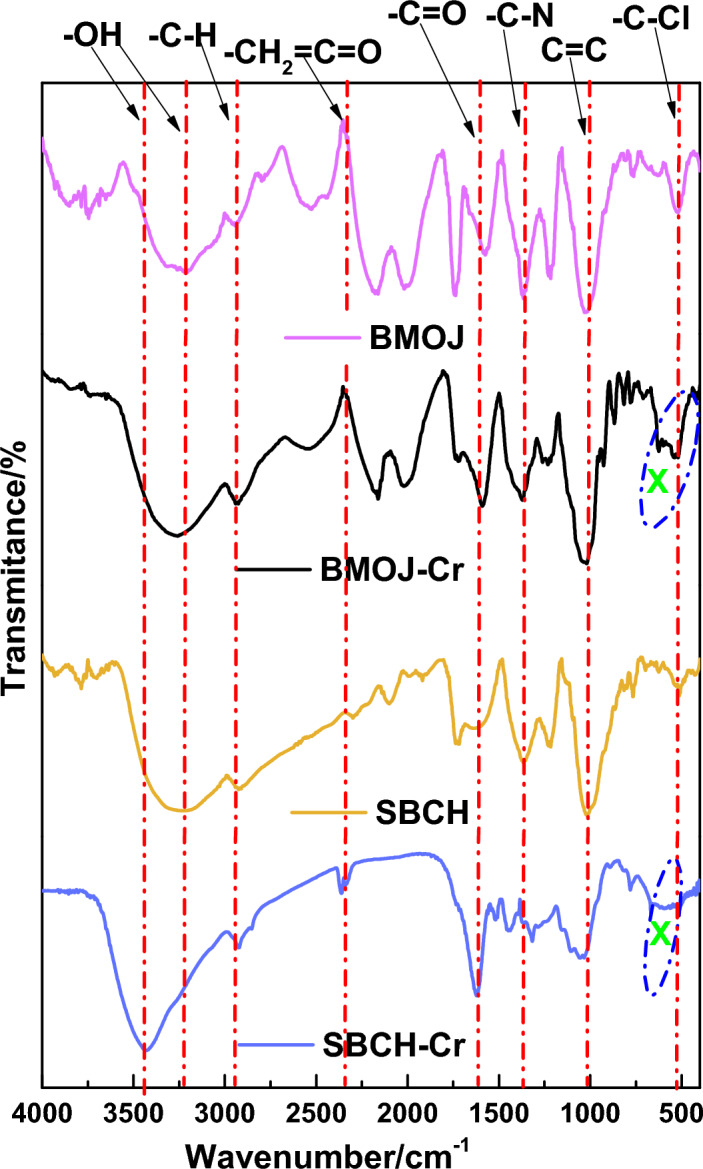
The FTIR spectra of SBCH, SBCH-Cr, BMOJ and BMOJ-Cr.

The FTIR spectra acquired for SBCH, SBCH-Cr, BMOJ and BMOJ-Cr seemed qualitatively comparable (see Fig. 2), with multiple common absorption bands, but with slight changes in relative intensities of the peaks. The following were the wavenumbers that are associated with the major absorption peaks of the pristine and chromium-loaded adsorbents. Bands at 3403 cm^−1^, 2909 cm^−1^, 2353 cm^−1^, 1631 cm^−1^, 1492 cm^−1^ and 1029 cm^−1^ (spectra were attributed to -OH of water, alcohol, phenols, –CH of alkane groups, O-containing groups such as lactonic and anhydride groups, C=O stretching mode, –C–N stretching mode, and C=C stretching mode, respectively^80^. The implication of *Allium cepa* juice extract on the surface of the pristine biochar was noticed with the creation of new peaks, shift in bands and enhanced intensity of existing peaks in the spectrum of the modified biochar. On the other hand, Fig. 2 revealed enhanced peak intensity (for bands 3403 and 605 cm^−1^) for spent adsorbents (SBCH-Cr, and BMOJ-Cr) when compared to the spectra of the pristine adsorbents (SBCH and BMOJ). This further justifies the interaction of chromium species with the surface of the SBCH-Cr, and BMOJ-Cr.

**Figure 3:**
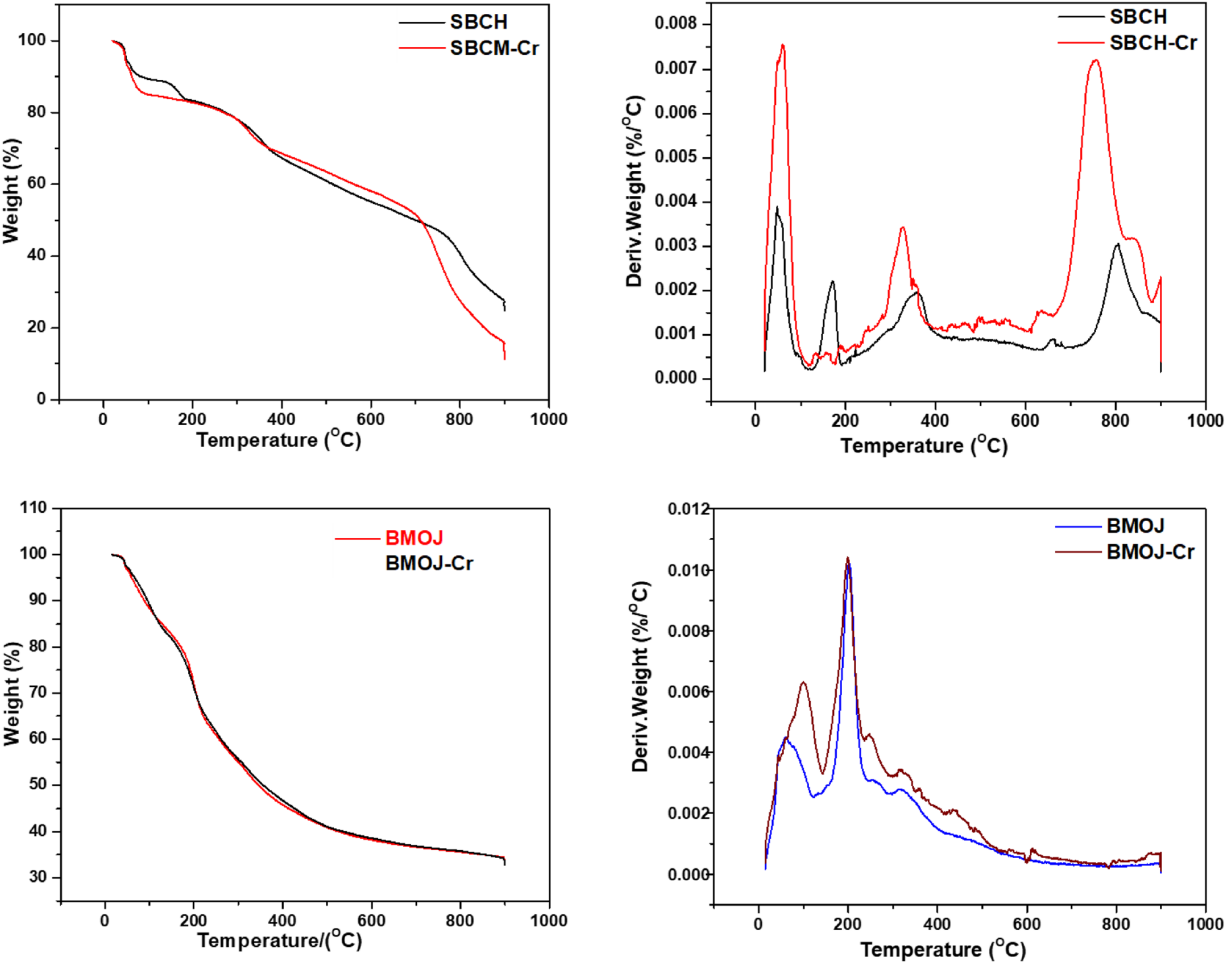
Thermogravimetric analysis curves for the natural SBCH, SBCH-Cr, BMOJ and BMOJ-Cr.

The thermogravimetric analysis curves for SBCH, SBCH-Cr, BMOJ and BMOJ-Cr are shown in Fig. 3. In the first stage (20–150 °C), about 9% and 18% weight loss was observed for SBCH and SBCH-Cr, respectively. In the same temperature range, about 15% weight loss was noticed for BMOJ and BMOJ-Cr. The weight loss within 20–150 °C is attributed to the volatilization of molecular-bound water. The emissions of volatile organic compounds may be responsible for the thermal degradation observed in the second stage within 150–250 °C^81^. When the pyrolytic temperature increased from 300 to 900 °C, a sharp loss in weight was noticed for both as-prepared and spent adsorbent. The loss of weight within this temperature range could be ascribed to the primary composition of the biomass used for biochar fabrication. The temperature ranges of hemicellulose, cellulose, and lignin breakdown were described as 210–320, 315–400, and 150–900 °C, respectively^82^. Thus, the 43.5% and 55.3% weight loss observed SBCH and SBCH-Cr within 340–900 could be attributed to cellulose, and lignin decomposition. Similar observations were made for BMOJ and BMOJ-Cr with 30% weight loss. The derivative mass-loss curve of the chromium-loaded adsorbent demonstrated more endothermic peaks within the range of investigated temperature. Spent adsorbents were notice to induce a slight shift in temperature bands of the peaks. This reflects the fixation of the chromium ions to the surface of the adsorbents.

**Figure 4:**
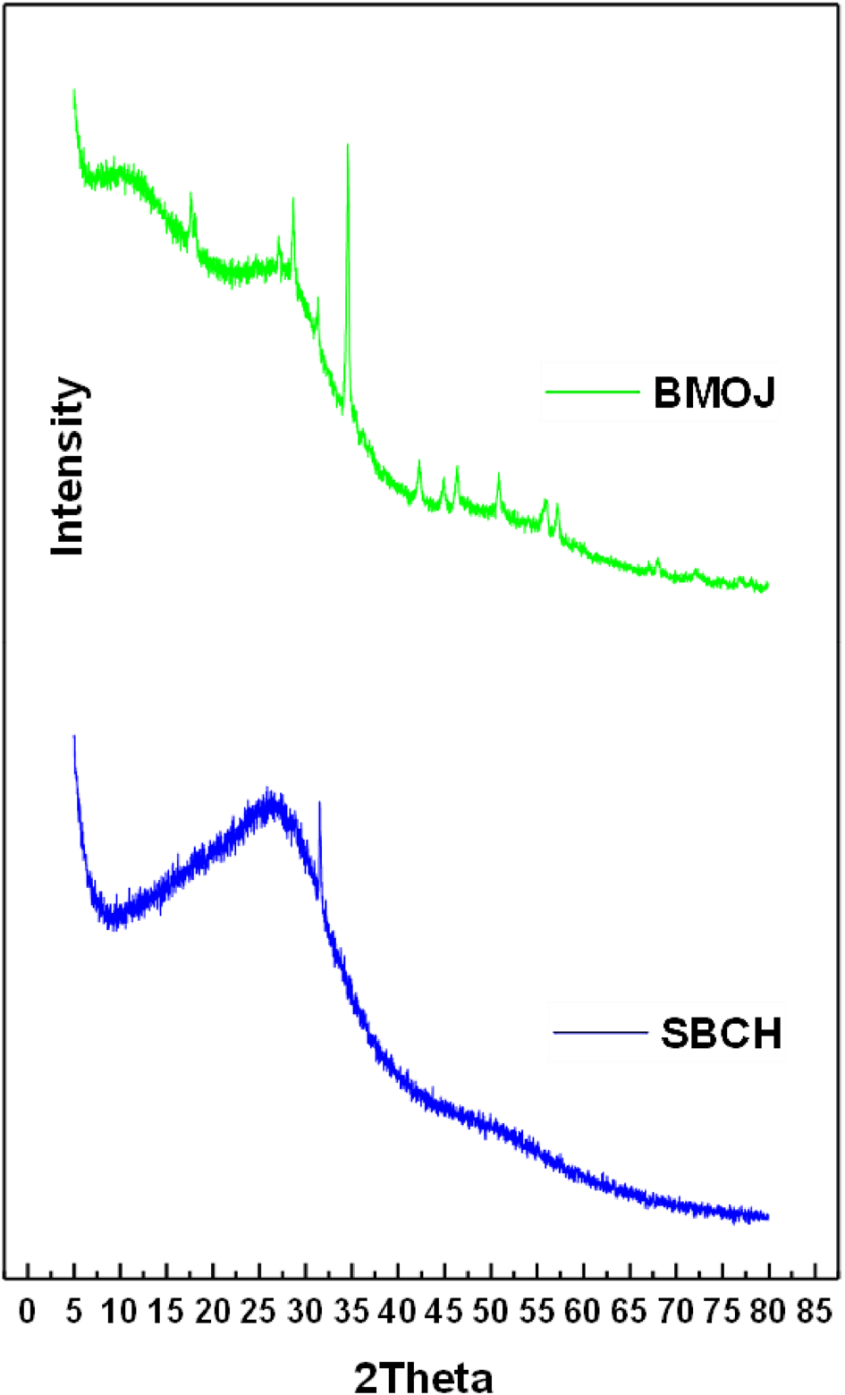
XRD pattern of SBCH and BMOJ.

The crystal structures of SBCH and BMOJ were investigated using the powder XRD. As illustrated in Fig. 4, the diffraction pattern at 2θ = 23.5°–30° is attributed to the crystal plane index C(0 0 2), which corresponds to the parallel and azimuthal orientation of the aromatic and carbonised structure^83^. The diffraction pattern acquired for SBCH exhibited a broad peak at 2θ = 47.5°–52.5° that is ascribed to C(1 0 0) diffractions of graphitic and hexagonal carbons^84^. The BMOJ showed a similar pattern to SBCH, except for a slight shift in peak the formation of new peaks. This shows that the modification step did not deform the crystallinity and the structural composition of the biochar.

**Figure 5:**
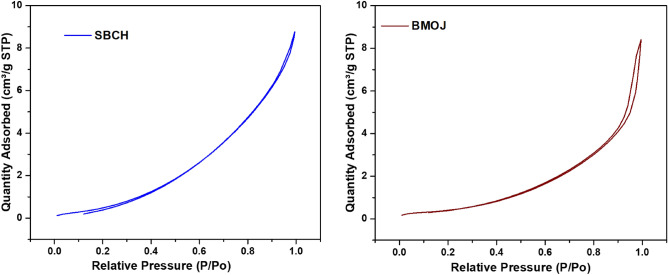
N_2_ adsorption–desorption isotherms SBCH and BMOJ.

The Barrett-Joyner-Halenda (BJH) pore-size distribution and the N_2_ adsorption–desorption isotherm approach were used to evaluate the surface characteristics of BMOJ and SBCH. The BET plots of BMOJ and SBCH reflected the type III physisorption isotherm (see Fig. 5). As a result, BMOJ and SBCH maintained larger micropores, wider pore size distributions, narrower mesopores, and excellent uptake capacity. Meanwhile, the minor hysteresis loop in the N_2_ adsorption–desorption isotherm at a relative pressure of 0.9 < P/P0 < 1 suggests capillary condensation, indicating the dominance of a mesoporous structure. BMOJ and SBCH had BET surface areas of 4.478 m^2^ g^−1^ and 0.8787 m^2^ g^−1^, respectively (see Table 3). The BET surface area acquired for the modified adsorbent was in good agreement with the report of previous authors^85–87^. The addition of *Allium cepa* juice extract to biochar was found to increase the pore diameter and surface area of BMOJ. As a result, the simple pore capture of chromium ions by BMOJ may improve the removal of hexavalent chromium from water.

**Table 3:** Textural properties of SBCH and BMOJ.

Adsorbents	Surface area/m^2^ g^−1^	Pore volume/cm^3^ g^−1^	Pore diameter/nm	pH_PZC_
SBCH	0.8787	0.009472	5.0841	6.04
BMOJ	4.478	0.006335	5.6592	5.74

**Figure 6:**
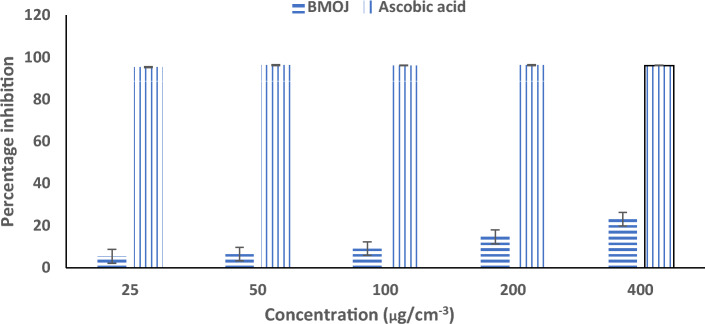
Antioxidant activity of BMOJ (DPPH).

**Figure 7:**
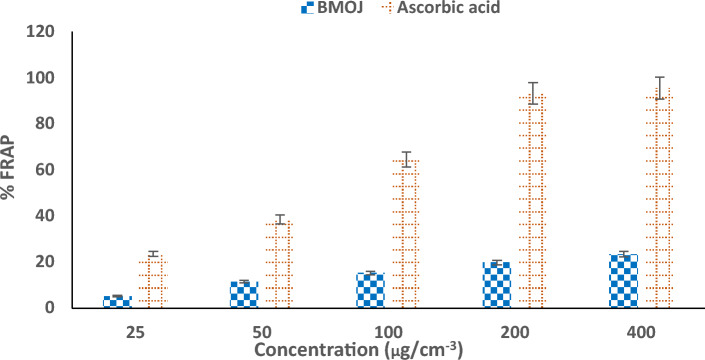
Ferric-reducing antioxidant power of BMOJ.

Antioxidants are chemical components that are utilized to halt oxidation reactions by preventing the creation of free radicals^88^. This class of chemicals also shield organic matter from nascent oxygen, which is damaging to biomolecules such as lipids, enzymes, proteins, and amino acids and can cause cell death^89,90^. The DPPH assay measures the potential to destroy natural macromolecules and phytochemicals; specifically, the presence of phenolic compounds in plant extract functions as an antioxidant agent. On the other hand, the ferric reducing antioxidant power (FRAP) assay is a standard electron transfer-based technique that evaluates the reduction of ferric ion (Fe^3+^)-ligand complex by antioxidants in an acidic medium to the intensely blue-coloured ferrous (Fe^2+^) complex^91^. The antioxidant activity of BMOJ was evaluated using DPPH and FRAP assay. Figures 6 and 7, showed that BMOJ has a moderate antioxidant activity when compared to standards (ascorbic acid). The antioxidant performance of BMOJ increased with increasing sample concentration in a dose-dependent manner. Meanwhile, the presence of secondary metabolites (phenolic chemicals) in the plant extract serves as antioxidant agents in the juice extract. Considering the capacity of BMOJ to reduce Fe^3+^ to Fe^2+^, suggests that the uptake of Cr (VI) by BMOJ may be attributed to the reduction of Cr (VI) to Cr(III).

### Influence of pH

**Figure 8:**
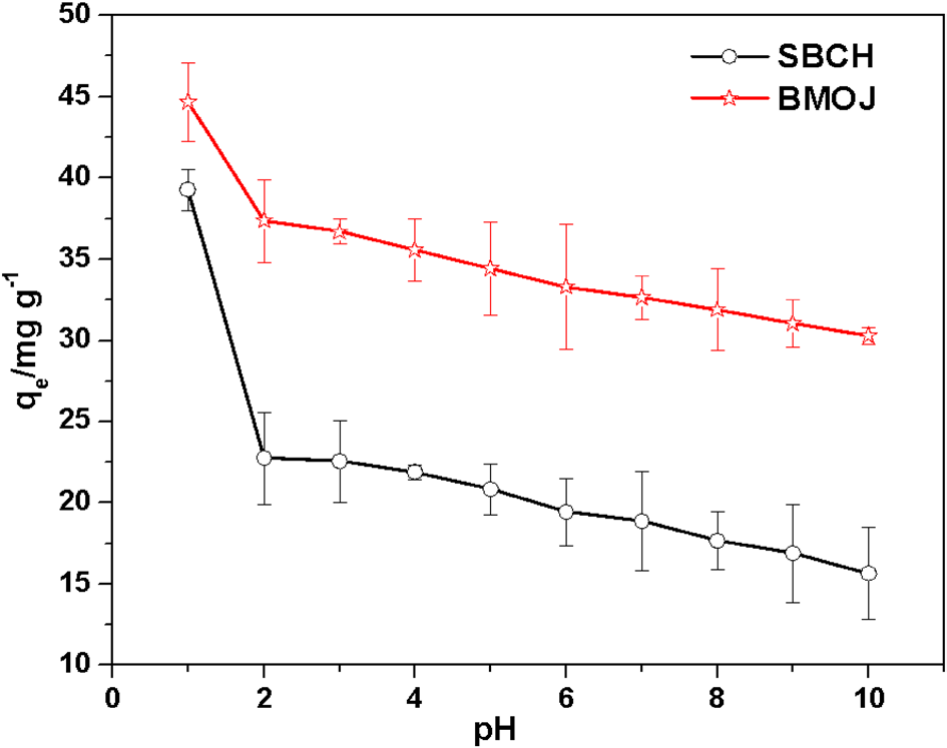
The influence of pH on the removal capacity of Cr (VI) by SBCH and BMOJ [conditions: 25 cm^3^ of 100 mg dm^−3^ Cr (VI), 180 min contact time, 0.05 g of dosage, agitation speed 150 rpm, temperature 25 °C].

**Figure 9:**
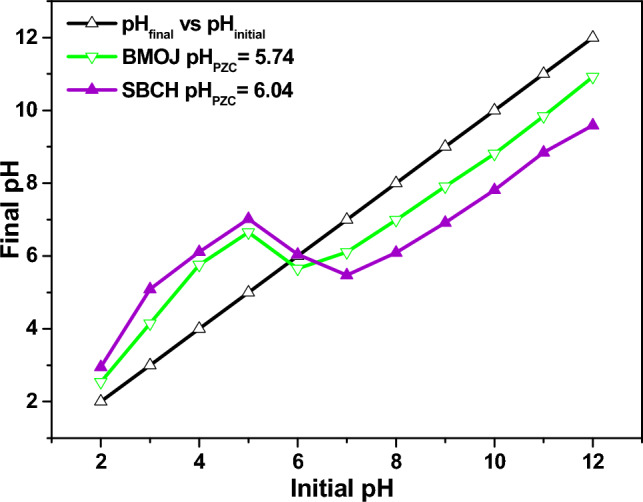
pHpzc plots of SBCH and BMOJ.

The potential of Cr (VI) adsorption by SBCH and BMOJ was noticed to remarkably decreased as the pH increased (see Fig. 8). At pH 2 the uptake capacity of SBCH and BMOJ reached 23.55 mg g^−1^ and 37.69 mg g^−1^, respectively. This implies that acidic environments were favourable for the elimination of Cr (VI) from the aqueous phase by SBCH and BMOJ. A lower solution pH condition (pH < 1.5) was more likely to damage the surface SBCH and BMOJ, hence, pH 2 was selected as the optimum pH for further study. Since the pH_PZC_ of SBCH and BMOJ were 6.04 and 5.74, respectively (see Fig. 9). Solution pH lower than the predetermined pHp_ZC_ of the adsorbents could make a more positive charge at the surface of SBCH and BMOJ. The positively charged adsorbents will interact more with oxyanions (HCrO_4_^−^ and Cr_2_O_7_^2−^ and CrO_4_^2−^) via electrostatic interactions. This observation was consistent with the report for the adsorption of Cr (VI) using other types of adsorbent^92–94^.

### Contact time

**Figure 10:**
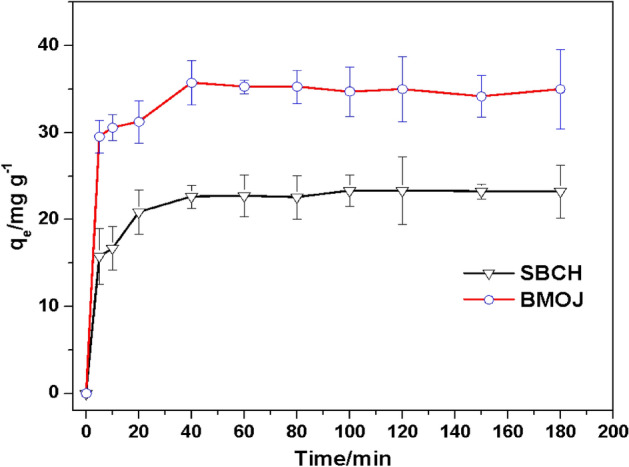
Effect of contact time on the adsorption of Cr (VI) unto SBCH and BMOJ [conditions: 25 cm^3^ of 100 mg dm^−3^ Cr (VI), solution pH of 2, 0.05 g of dosage, agitation speed 150 rpm, temperature 25 °C].

As shown in Fig. 10, the sequestration of Cr (VI) from the aqueous phase by SBCH and BMOJ occurred in two stages. In the first stage, rapid adsorption of chromium(VI) by SBCH and BMOJ was noticed with a removal potential of 2.85 and 31.46 mg g^−1^, respectively at 20 min. The rapid adsorption of Cr (VI) within the first 20 min was attributable to the significant initial concentration difference between Cr (VI) in solution and the amount of Cr (VI) on the surface of the adsorbents, also the high amount of available unoccupied sites on the surface of SBCH and BMOJ at the initial stage may be responsible for the fast uptake of Cr (VI). The subsequent stage was characterized by a slower continuous adsorption process. After the first 20 min, there was a progressive increase that eventually levelled off after 40 min. At 40 min, there was no significant increase or decrease in the adsorption of Cr(VI), indicating equilibration due to adsorption site saturation. However, 180 min was used for the subsequent experiment to ensure equilibrium attainment. Meanwhile, the results obtained were consistent with other studies^95–97^.

### Adsorption kinetics

**Figure 11:**
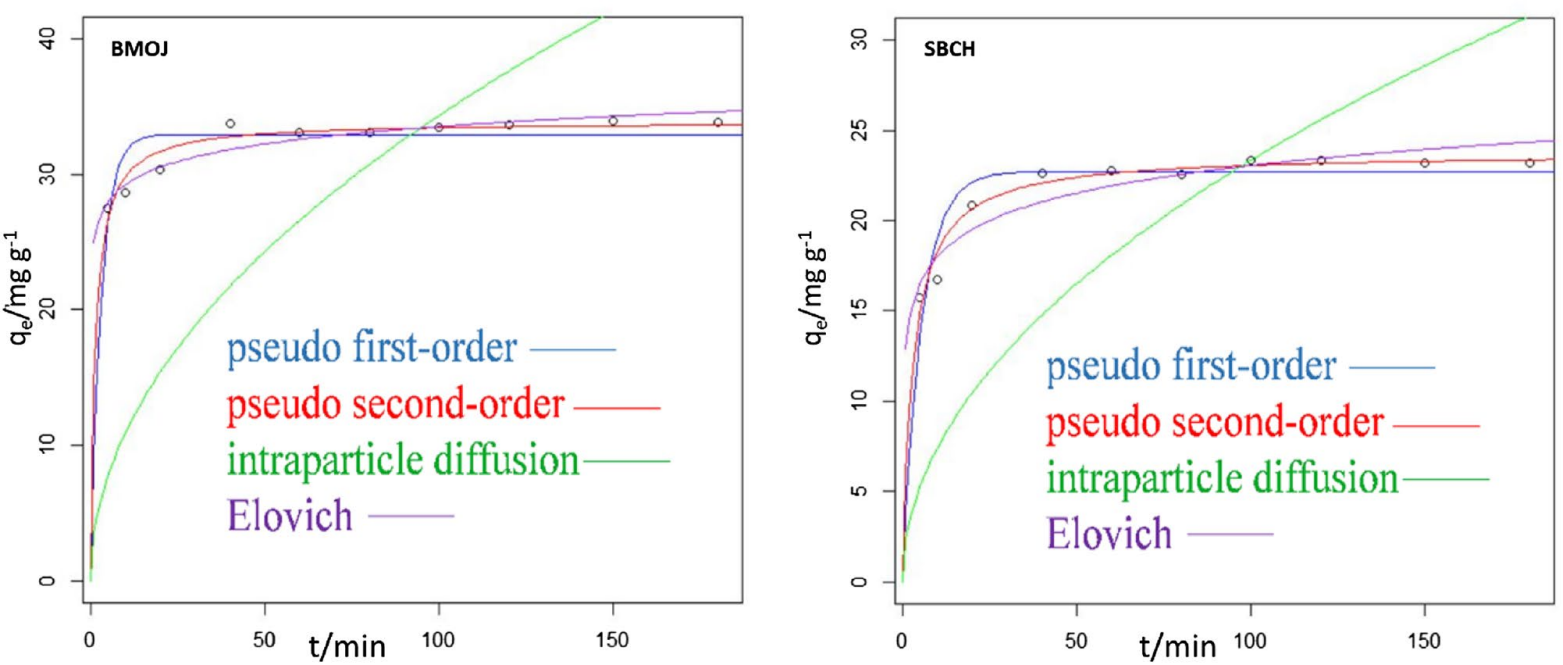
Plots of kinetics models fitted to the experimental data for the adsorption of Cr (VI) onto SBCH and BMOJ.

The kinetics data obtained from the agitation period experiment were further examined by the Elovich, pseudo-first-order (PFO), Intraparticle diffusion (ID) and pseudo-second-order (PSO) models. According to the PFO model, the rate of change of adsorbate uptake with respect to time is proportional to a change in equilibrium concentration and amount of uptake relative to time^98,99^. Meanwhile, the PSO model assumes that chemisorption is the rate-determining step that drives the adsorption process^100–102^. On the other hand, the Elovich kinetic model tends to assess the adsorption rate, which reduces rapidly as adsorbate concentration increases^103^. Finally, the Weber-Morris intraparticle diffusion model is sectioned into three phases and is used to explain the diffusion mechanism during adsorption. The first phase is characterized by quick adsorption on the exterior surface, while the second phase is characterized by intraparticle diffusion, followed by sluggish equilibrium in the third phase^104^. The non-linearized plots comparing the tested models are displayed in Fig. 11. Kinetic parameters such as *k*_1_, *k*_2_, *q*_e_, α, β, and K_id_ as well as the sum of square residuals (SSR) and residual square errors (RSE) were also estimated and listed in Table 4. The kinetic model with the least SSR value was assumed to fit the experimental data best. A comparison of the four kinetic models used for this study, presented PSO as the model to best describe the adsorption of Cr (VI) onto SBCH (SSR = 3.874) and BMOJ (SSR = 5.245). Additionally, the q_max_ estimated for the PFO model was significantly different from the experimental *q*_exp_, signifying that the PFO model lacks the capacity to well describe the uptake of Cr (VI) onto SBCH and BMOJ. Owning to the close SSR value of Elovich model 9.101 (SBCH) and 5.305 (BMOJ), the adsorption of Cr (VI) onto the SBCH and BMOJ were heterogeneous or multi-mechanism driven. Meanwhile, a greater α value relative to β may also indicate fast adsorption in the early stages^105^. This further justify the observation made in the contact time experiment. To access clarity of the Cr (VI) uptake by SBCH and BMOJ, IPD model was employed. The mechanism of adsorption is multi-step regulated for a linear plot of q_t_ versus t^1/2^ in which the linear plot does not pass through the origin. If the linear plot passes through the origin, intraparticle diffusion of the Cr (VI) ions into the pores of the SBCH and BMOJ will be the rate-determining step. A non-zero intercept was anticipated for SBCH and BMOJ based on the data, implying that more than one rate-limiting step was responsible for the removal of Cr (VI) by SBCH and BMOJ.

**Table 4:** Estimated parameters of Elovich, intra-particle diffusion pseudo-first order and pseudo-second order kinetic models for Cr (VI) adsorption onto SBCH and BMOJ at different time intervals.

Model	Parameter	SBCH	BMOJ
Experimental	q_exp_/mg g^−1^	23.21	33.86
Pseudo first order	K_1_/min^−1^	0.1812	0.3193
	q_eq_/mg g^−1^	22.7283	32.955
	SSR	13.04	19.9
	RSE	1.277	1.577
Pseudo second order	K_2_/g mg^−1^ min^−1^	0.01398	0.02135
	q_eq_/mg g^−1^	23.75124	33.9107
	SSR	3.874	5.245
	RSE	0.6959	0.8097
Intraparticle diffusion	K_id_/mg g^−1^ min^−0.5^	2.335	3.436
	l/mg g^−1^	–	–
	SSR	492.5	1362
	RSE	7.398	12.3
Elovich	α/mg g^−1^ min^−1^	12.908	24.923
	β/g mg^−1^	2.202	1.872
	SSR	9.101	5.305
	RES	1.067	0.8143

### Effect of adsorbent dosage on Cr (VI) adsorption

**Figure 12:**
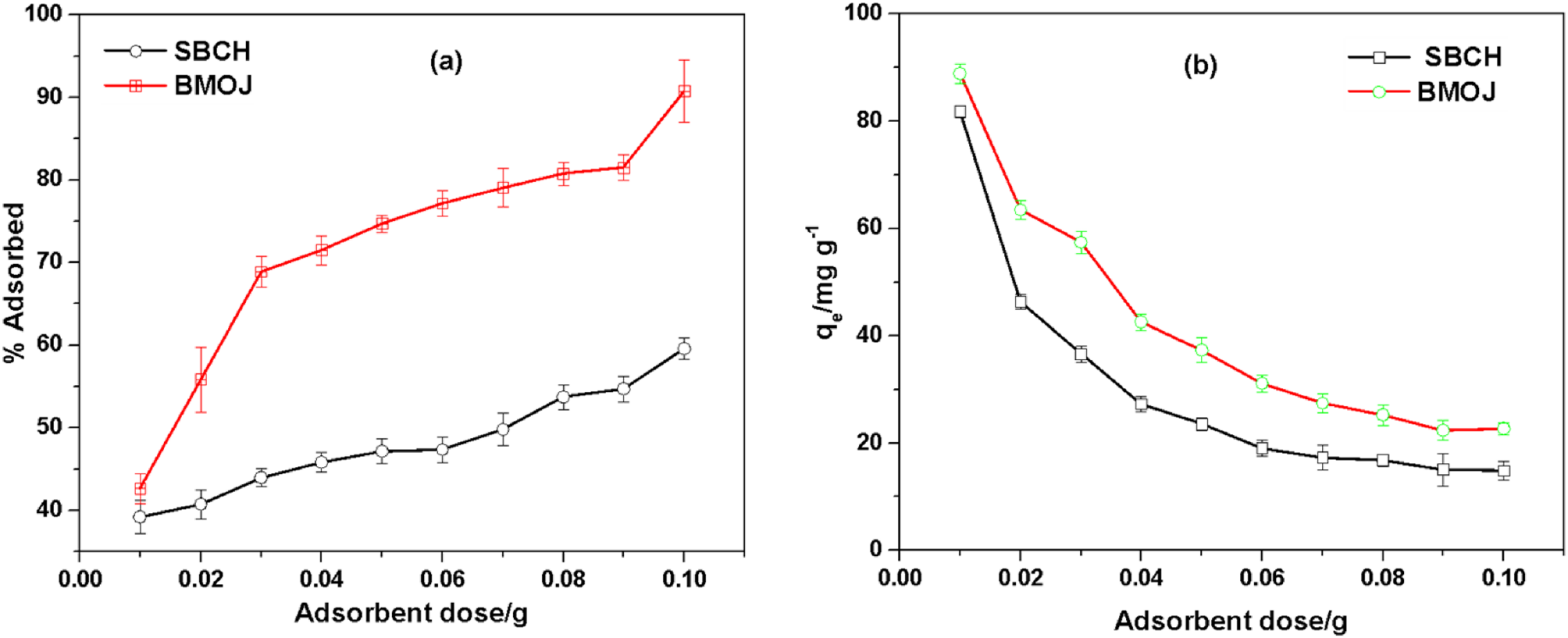
Effect of adsorbent dose on the adsorption of Cr (VI) onto SBCH and BMOJ (**a**) Adsorption efficiency (**b**) adsorption capacity [conditions: 25 cm^3^ of 100 mg dm^−3^ Cr (VI), solution pH of 2, 180 min contact time, agitation speed 150 rpm, temperature 25 °C].

As shown in Fig. 12, the removal efficiencies of Cr (VI) by SBCH and BMOJ were enhanced when the adsorbent dose increased. The adsorption efficiencies of Cr (VI) increased from 38.14 to 57.61% for SBCH and 43.27 to 92.35% for BNOJ when the adsorbent dose increased from 0.01 to 0.1 g. This could be attributed to a huge number of unoccupied higher adsorption sites with larger surface areas^106^. However, the adsorption capacity of Cr (VI) decreased with an increase in adsorbent dose. The removal potential decreased from 82.04 to 18.58 for SBCH and 89.57 to 24.74 mg g^−1^ for BMOJ, respectively (see Fig. 12). This phenomenon can be associated with the increased unsaturated adsorption site caused by the agglomeration of the adsorbent with increased dosage^27,107,108^.

### Adsorption isotherm studies

**Figure 13:**
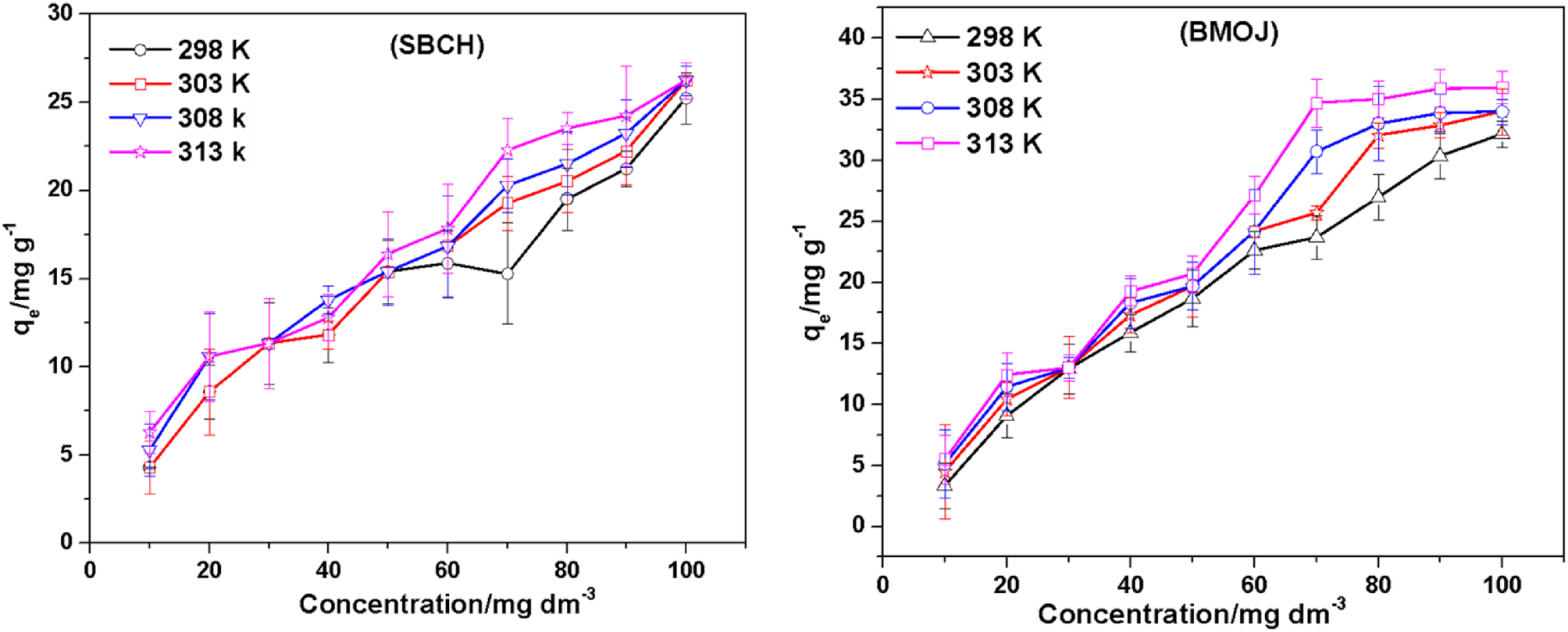
The implication of initial Cr (VI) concentration and solution temperature on the uptake capacity of OJMW and MWCNTs for Cr(VI) [conditions: 25 cm^3^ of Cr (VI) solution, pH of 2, 180 min contact time, agitation speed 120 rpm, 0.05 g of adsorbent dose, temperature of 25 °C].

Adsorption isotherms are well recognized for being frequently used to illustrate the physical/chemical adsorption of a dynamic equilibrium process, which could offer essential information via the prediction of adsorption potential of adsorbents using optimized adsorptive parameters such as optimum pH, initial absorbate concentration, optimum contact period, solution temperature of adsorption, and the optimum adsorbent dosage^109^. In order to access a deeper understanding of the interaction of Cr (VI) ions with BMOJ and SBCH during the adsorption process, the influence of initial concentration on the uptake of Cr (VI) by BMOJ and SBCH was studied at initial Cr (VI) concentrations ranging from 10 to 100 mg dm^−3^. It was observed that the increase in initial Cr (VI) concentrations resulted in an increase in the uptake of adsorption capacity from 5 to 35 mg g^−1^ for BMOJ and 5.1 to 25 mg g^−1^ for SBCH, respectively (see Fig. 13). This could be due to an increase in the concentration gradient that induces a motive force resulting in enhanced mass transfer that tends to facilitate adsorbate penetration into the adsorbent's interior pores and layers. However, increasing the solution temperature also increased Cr (VI) uptake, which might be due to the creation of additional adsorption sites or the expansion of the adsorbent pores, resulting in further Cr (VI) onto BMOJ and SBCH. Freundlich and Langmuir isotherm models were created using experimental data acquired from adsorption equilibrium studies. The Langmuir isotherm was founded on the monolayer adsorption principle, with the assumption that all sorbent adsorption sites were comparable and independent. Meanwhile, the Freundlich isotherm was employed to assess the relationship between the equilibrium concentration of Cr (VI) and the adsorption capacity of porous BMOJ and SBCH, as well as to investigate the interaction of Cr (VI) ions with the surface of BMOJ and SBCH. The isotherm parameters determined from the nonlinear fitting equation are shown in Table 5. In the system of Cr (VI) adsorption onto porous BMOJ and SBCH, the SSR of the Freundlich isotherm model was lower than that of the Langmuir isotherm model, suggesting that the adsorption process followed the Freundlich model rather than the Langmuir model. Because the nanopores on the surface of BMOJ and SBCH supplied numerous adsorption sites in three dimensions for Cr (VI) uptake, hence, the uptake of Cr (VI) onto BMOJ and SBCH was mostly due to a multi-molecular layered adsorption. Meanwhile, the secondary mode of eliminating Cr (VI) was monolayer adsorption, which occurred mostly on the smooth areas on the surface of BMOJ and SBCH that lacked nanopores, this can be observed in the SEM micrograph (see Fig. 1). The n and k_F_ values are presented in Table 5. It was widely understood that a value of 2 ≤ n ≤ 10 indicated easy adsorption, a value ≤ 1 n < 2 suggested a moderate uptake process, and finally, a value of n < 1 indicated hard adsorption^110^. The value of n in BMOJ was far less than 1 and the n value estimated from SBCH was ≈ 1, indicating that the uptake of Cr (VI) onto BMOJ and SBCH was a hard process (suggesting constrained adsorbate removal as the adsorption process proceeds). Furthermore, a comparison of the removal capacity obtained for BMOJ and SBCH with other adsorbent that were employed for Cr (VI) removal showed that BMOJ is a promising adsorbent for the sequestration of hexavalent chromium (see Table 6).

**Table 5:** Isotherm parameters for Cr (VI) adsorption onto BMOJ and SBCH.

Adsorbent	Isotherm	Parameters	Temperature			
			295 K	303 K	310 K	318 K
BMOJ	Freundlich	K_F_	0.56683	0.56674	0.56884	0.57104
		n	1.01724	1.01685	1.01722	1.01745
		SSR	17.32697	20.5098	23.56383	28.82341
	Langmuir	qm	28.4237	32.53346	34.64432	37.3812
		b	0.01	0.01	0.01	0.01
		SSR	1795.221	1859.825	1878.504	2010.741
SBCH	Freundlich	K_F_	0.50621	0.41832	0.72632	0.87759
		n	1.01977	0.98261	1.0576	1.08802
		SSR	48.48594	21.20268	72.67359	126.58032
	Langmuir	qm	26.33051	26.71673	25.66215	25.76857
		b	0.01	0.01	0.01	0.01
		SSR	854.762	1049.466	1282.595	1518.097

**Table 6:** A comparison of the Langmuir maximum adsorption capacities, q_m_, for the adsorption of Cr (VI) ions by different adsorbents.

Adsorbents	pH	Temperature (°C)	q_m_/mg g^−1^	References
Chitosan	4.0	25	154.0	^111^
Wheat bran	2.0	–	35.00	^112^
Indica seed (TS)	6.0	50	50.00	^113^
Fe_3_O_4_	3.0	33	3.46	^114^
Soya cake	< 1	20	0.0003	^115^
Spent activated clay	2.0	24	1.420	^14^
MnFe_2_O_4_	3.0	33	3.210	^114^
Norit carbon (oxidized)	2.0	30	10.60	^116^
Raw rice bran	5.0	25	0.070	^117^
Lignin	2.0	25	5.200	^118^
Norit carbon (oxidized)	3.7	40	53.00	^119^
Oxidized-MWCNTs	2.9	20	3.040	^120^
BMOJ	2.0	22	37.38	This study
SBCH	2.0	22	25.77	This study

### Thermodynamic study

The feasibility and nature of the adsorption reaction were determined by calculating thermodynamic parameters such as enthalpy change (ΔH), Gibbs free energy change (ΔG) and entropy change (ΔS)^121–123^. The following equations were used to estimate these parameters.

5 $$ \Delta G^\circ = - RT\ln K $$

6 $$ \ln K = - \frac{\Delta H^\circ }{{RT}} + \frac{\Delta S^\circ }{R} $$

where T, R and K denote temperature, gas constant and temperature-dependent equilibrium constant. When the initial Cr (VI) concentration and solution temperature were varied from 10 to 100 mg dm^−3^ and 298 to 313 K, respectively. The thermodynamics parameters (ΔS and ΔH) of the adsorption process were calculated from the intercept and slope of the curve relating InK to 1/T. The ΔG° values at all temperatures were negative, indicating that the adsorption of Cr (VI) onto BMOJ and SBCH was spontaneous. Meanwhile, the estimated ΔH° values were positive and negative for both BMOJ and SBCH, which confirmed that the removal mechanism was indeed endothermic and exothermic respectively. On the other hand, the calculated ΔS° values were positive for BMOJ and SBCH, respectively. This confirmed the increase of the randomness of the solid–liquid interface during the adsorption process (see Table 7).

**Table 7:** Thermodynamic parameters estimated for the uptake of Cr (VI) onto BMOJ and SBCH.

Adsorbents	T/K	ΔG°/kJ mol^−1^	ΔH/kJ mol^−1^	ΔS/J K^−1^ mol^−1^
BMOJ	298	− 13.9986	13.74	93.258
	303	− 14.5737		
	308	− 14.9752		
	313	− 15.4161		
SBCH	298	− 13.8091		
	303	− 14.0774	− 16.22	40.96
	308	− 14.2066		
	318	− 14.448		

### Regeneration and reusability study

**Figure 14:**
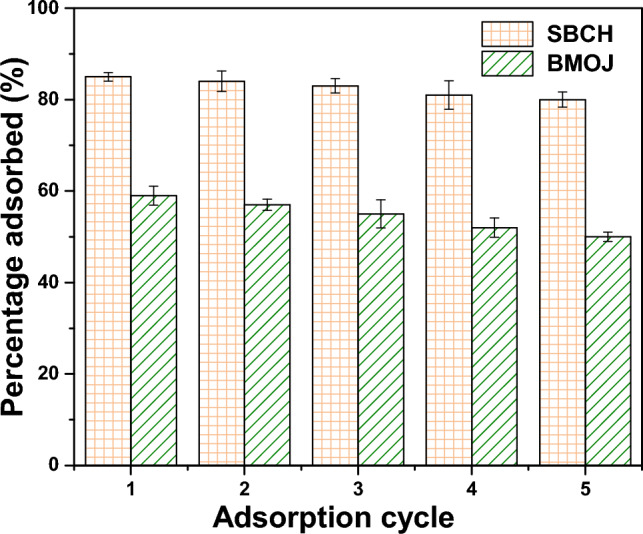
The Cr (VI) adsorption efficiency of BMOJ and SBCH after different cycles.

Adsorbent recyclability is critical, especially in terms of energy usage, economic implications, and environmental safety. BMOJ and SBCH containing Cr (VI) were desorbed using 1 mol dm^−3^ sodium hydroxide. Figure 14 depicts the outcomes of five reused cycles. The recyclability of SBCH appears to be decreased when compared to BMOJ containing Cr (VI) ions. After five cycles, the effective adsorption rate of SBCH containing Cr (VI) ions was 49.7%, while 80.5% was obtained for BMOJ. The analysis revealed that BMOJ can be reused for five consecutive cycles, hence, the economic implication of BMOJ is an added value to this water treatment agent, as such can be scaled up for industrial application.

### Removal mechanism of Cr (VI) by BMOJ

**Figure 15:**
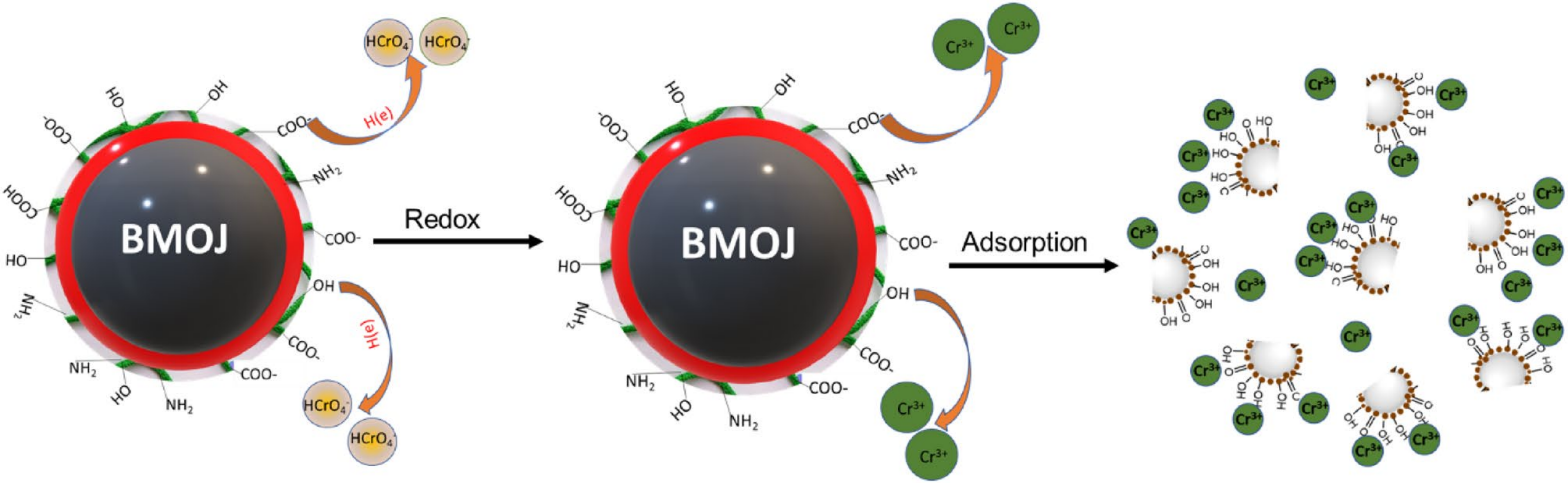
Mechanisms of interaction that could exist between BMOJ and Cr (VI).

A mechanism responsible for the adsorptive removal of Cr (VI) by BMOJ at solution pH 2 is suggested, and the schematic model of the removal is shown in Fig. 15. The electrostatic interaction of the oxyanion species of Cr (VI) with the protonated surface of BMOJ at pH 2 facilitates Cr (VI) engagement with BMOJ. The FTIR showed essential peaks (hydroxyl and carbonyl) on the surface of BMOJ that are anticipated to enhance Cr (VI) uptake. There are possible alternative methods for removing Cr (VI). The first possible reaction path could be via ion exchange and hydrogen bonding, but in the second pathway, Cr (VI) was absorbed to the inner part via intraparticle diffusion/pore entrapment, followed by a pre-adsorption and reduction of Cr (VI) into its Cr(III) state. As a result, the reduction and anionic adsorption via the electrostatic interaction model may adequately explain the mechanism behind the uptake of Cr (VI) by BMOJ.

## Conclusion

In summary, new adsorbents (BMOJ and SBCH) were successfully synthesized and used in the aqueous adsorption of Cr(VI). The optimum capacity for Cr (VI) adsorption is 35.95 mg g^−1^ and 26.22 mg g^−1^ for BMOJ and SBCH, respectively. The uptake of Cr (VI) by BMOJ and SBCH, is influenced by adsorbate pH, contact time and dosage. Meanwhile, optimal adsorptive conditions were developed, including pH 2, 0.05 g adsorbent dosage, 180 min contact period, and 100 mg dm^−3^ starting concentration. The adsorptive removal of Cr (VI) by BMOJ and SBCH was best described by the pseudo-second-order kinetic model and Freundlich model, demonstrating a chemical process for adsorption. Thermodynamic studies revealed that the elimination of Cr (VI) by BMOJ and SBCH was spontaneous. The surface characteristics of SBCH were generally improved by *Allium cepa* juice extract, with a higher adsorption of Cr (VI) on the modified adsorbent (BMOJ). After five cycles of regeneration and reuse, the water treatment agents demonstrated Cr (VI) uptake of 49.7% and 80.5% for SBCH and BMOJ, respectively. Finally, *Allium cepa* juice extract-coated biochar can be considered fit for the treatment of Cr (VI) contaminated water (Supplementary Information S1).

### Supplementary Information

Supplementary Information.

## Data Availability

The authors declare that all data generated and analysed are available within the manuscript [and its supplementary information file].
