# Supplementary material for: Preparation and characterization of Allium cepa extract coated biochar and adsorption performance for hexavalent chromium
Source: Sci Rep. 2023 Nov 27;13:20786. doi: 10.1038/s41598-023-48299-8 (PMC10682498; doi:10.1038/s41598-023-48299-8)

**Preparation and characterization of *Allium cepa* extract coated biochar and**

**adsorption performance for hexavalent chromium**

**James Friday Amaku and Raymond Taziwa**

Department of Applied Science, Faculty of Science Engineering and Technology, Walter Sisulu University, Old King William Town Road, Potsdam Site, East London 5200, South Africa.

* Correspondence: famaku@wsu.ac.za

a.

**Estimation of thermodynamics parameters (BMOJ)**


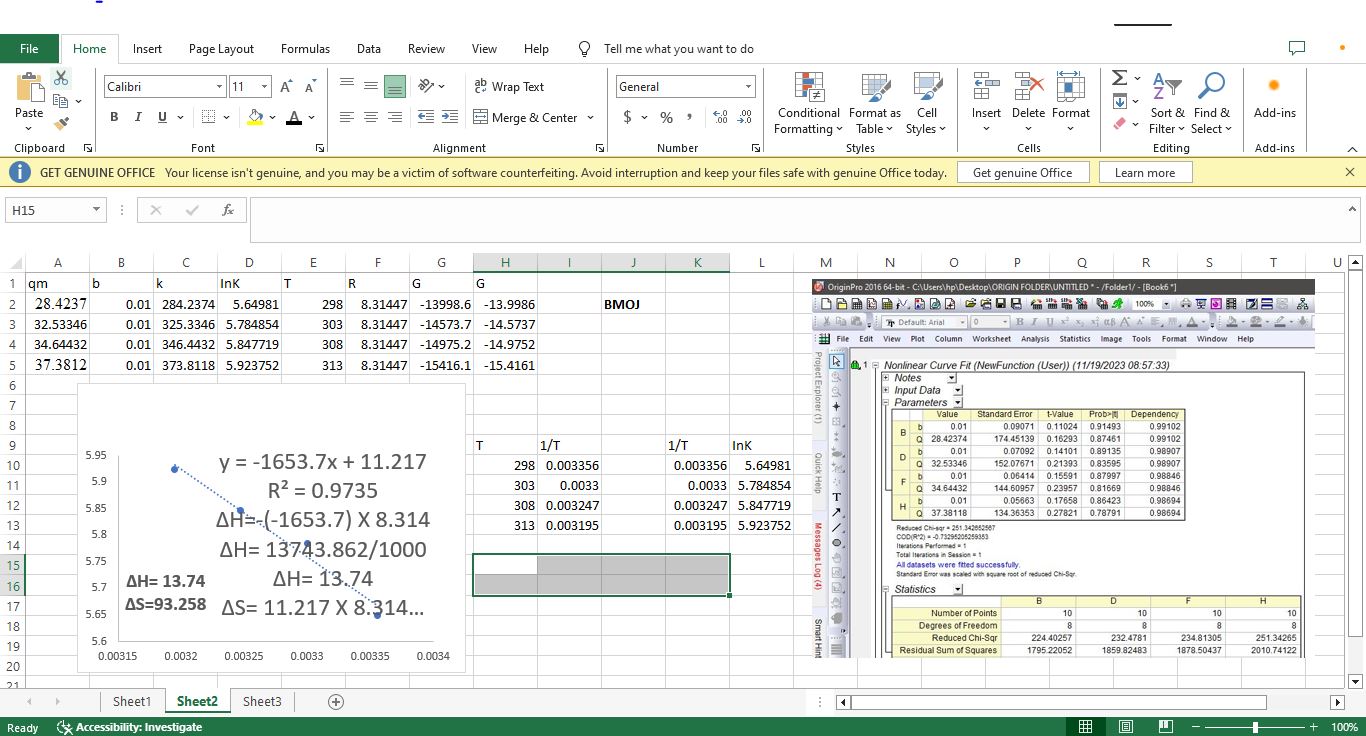


**Estimation of thermodynamics parameters (SBCH)**


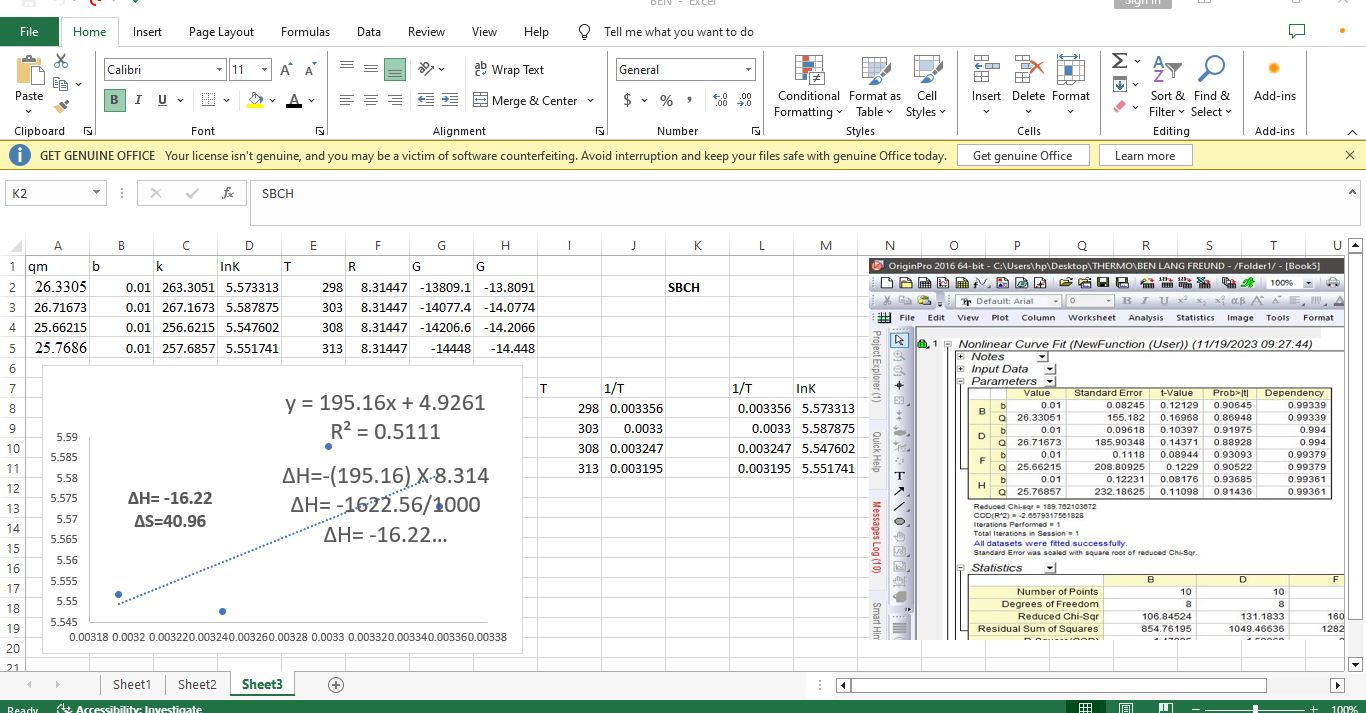

Supplement: Supplementary file 1 — Supplementary Information. [file 41598_2023_48299_MOESM1_ESM.docx]
